# Beta-Blocker Use in Older Hospitalized Patients Affected by Heart Failure and Chronic Obstructive Pulmonary Disease: An Italian Survey From the REPOSI Register

**DOI:** 10.3389/fcvm.2022.876693

**Published:** 2022-05-16

**Authors:** Vincenzo Arcoraci, Francesco Squadrito, Michelangelo Rottura, Maria Antonietta Barbieri, Giovanni Pallio, Natasha Irrera, Alessandro Nobili, Giuseppe Natoli, Christiano Argano, Giovanni Squadrito, Salvatore Corrao

**Affiliations:** ^1^Department of Clinical and Experimental Medicine, University of Messina, Messina, Italy; ^2^SunNutraPharma, Academic Spin-Off Company of the University of Messina, Messina, Italy; ^3^Department of Neuroscience, IRCCS Istituto di Ricerche Farmacologiche Mario Negri, Milan, Italy; ^4^Department of Health Promotion, Mother and Child Care, Internal Medicine and Medical Specialties “G. D'Alessandro”, PROMISE, University of Palermo, Palermo, Italy; ^5^Department of Internal Medicine, National Relevance and High Specialization Hospital Trust ARNAS Civico, Palermo, Italy

**Keywords:** COPD, heart failure, REPOSI register, clinical practice, beta-blockers

## Abstract

Beta (β)-blockers (BB) are useful in reducing morbidity and mortality in patients with heart failure (HF) and concomitant chronic obstructive pulmonary disease (COPD). Nevertheless, the use of BBs could induce bronchoconstriction due to β2-blockade. For this reason, both the ESC and GOLD guidelines strongly suggest the use of selective β1-BB in patients with HF and COPD. However, low adherence to guidelines was observed in multiple clinical settings. The aim of the study was to investigate the BBs use in older patients affected by HF and COPD, recorded in the REPOSI register. Of 942 patients affected by HF, 47.1% were treated with BBs. The use of BBs was significantly lower in patients with HF and COPD than in patients affected by HF alone, both at admission and at discharge (admission, 36.9% vs. 51.3%; discharge, 38.0% vs. 51.7%). In addition, no further BB users were found at discharge. The probability to being treated with a BB was significantly lower in patients with HF also affected by COPD (adj. OR, 95% CI: 0.50, 0.37–0.67), while the diagnosis of COPD was not associated with the choice of selective β1-BB (adj. OR, 95% CI: 1.33, 0.76–2.34). Despite clear recommendations by clinical guidelines, a significant underuse of BBs was also observed after hospital discharge. In COPD affected patients, physicians unreasonably reject BBs use, rather than choosing a β1-BB. The expected improvement of the BB prescriptions after hospitalization was not observed. A multidisciplinary approach among hospital physicians, general practitioners, and pharmacologists should be carried out for better drug management and adherence to guideline recommendations.

## Introduction

Chronic obstructive pulmonary disease (COPD) is a major cause of morbidity and mortality worldwide (1). COPD is a multisystem disease often associated with several concomitant conditions and, in particular, with cardiovascular diseases (CVD). Moreover, the gradual progression of COPD results from a combination of risk factors (2–5). Actually, COPD-related mortality is probably underestimated because of the difficulty to ascribe death to a single cause (6): approximately 30% of deaths of patients with COPD is attributable to CVD and, in particular, to myocardial infarction and heart failure (HF) (7). Moreover, according to the Cardiovascular Health Study, the prevalence of COPD is greater in patients affected by HF than in not affected subjects (8, 9).

Beta (β)-blockers (BBs) are widely prescribed β-adrenergic blocking agents, useful in reducing morbidity and mortality in patients with COPD and concomitant CVD (10–12). Nevertheless, β-blockade could induce bronchoconstriction and worsen lung function in patients with COPD (13). Despite a common mechanism of action, BBs have a different selectivity for receptor subtypes (14). In fact, only non-cardioselective BBs are related to a high risk of obstructive airway disease exacerbations (12, 15–17). In addition, the use of the non-selective BB carvedilol is associated with an increased risk of hospitalization for HF in patients affected by COPD and HF and also with drug discontinuation compared to cardiac selective β1-receptor antagonist use (i.e., metoprolol, bisoprolol, or nebivolol) (18). Otherwise, different studies reported that the use of cardioselective BBs does not significantly increase the risk of COPD exacerbation (19–22).

As a consequence, the European Society of Cardiology (ESC) and the Global Initiative for Chronic Obstructive Lung Disease (GOLD) guidelines suggest the safe and effective use of selective β1 BBs in patients with concurrent HF and respiratory diseases while strongly discouraging the use of non-selective BBs with vasodilating action like carvedilol (23, 24). Despite these evidences, few prescriptions of BBs were detected in patients affected by HF and concurrent COPD in multiple clinical settings; moreover, the pharmacological treatment with β1-selective BBs in this kind of patients appears to be underused (25–28), and a low adherence to clinical guidelines was observed (18).

The proper use of BBs, mainly in fragile patients such as older adults with HF and COPD, is essential even if the evidences are inadequate. The factors affecting BB prescription and selection are not well known. Additionally, few data on BB prescriptions are available, and Geriatric and Internal Medicine Departments represent a useful field of investigation to analyze older patients with comorbidities and in polypharmacy. Moreover, hospital admissions provide an opportunity to reevaluate treatments in patients with HF and COPD. In fact, a better familiarity with BBs and their contraindications by a hospital physician, in collaboration with a pharmacologist, could help identify appropriate preventive strategies for the management of these patients at discharge.

In the light of the above reasons, the aim of the study was to investigate the use and the appropriateness of different BBs in older patients affected by HF and HF/COPD, admitted to the internal medicine and geriatric wards and recorded in the REPOSI register.

## Materials and Methods

### Study Design and Data Collection

All data concerning patients were extracted from the REPOSI Registry database. REPOSI is a collaborative and independent registry of the Italian Society of Internal Medicine (SIMI), the IRCCS Mario Negri Institute for Pharmacological Research, and the Foundation of the IRCCS Cà Granda Maggiore Hospital in Milan. The introduction of the register was aimed at recruiting, monitoring, and evaluating older hospitalized patients aged 65 or more, admitted to 102 Italian internal medicine and geriatric wards. The register was established in 2008; information came from each single medical record and was collected every 2 years (29, 30). The main data collected include sociodemographic factors, laboratory data, comorbidities, and drug therapies. An encrypted code is used for each patient to comply with the law on the privacy of personal data. The study was conducted according to the guidelines of the Declaration of Helsinki and approved by the Ethics Committee of IRCCS Cà Grande Ospedale Maggiore Policlinico di Milano (approval number 43-2012).

For this study, all 4,713 patients recorded in the REPOSI Register between 2010 and 2016 were considered. Patients affected by HF and COPD were identified in accordance with the International Classification of Diseases 9th Revision (ICD-9-CM), as well as all other comorbidities. Drugs were classified according to ATC classification. The following clinical characteristics were evaluated: sex, age, BMI, indices of comorbidity and severity according to the cumulative disease assessment scales CIRS_C and CIRS_S (31, 32), mood disorders using the Geriatric Depression Scale (GDS) (33, 34), performance in basic activities of daily living, measured by the Barthel Index (BI) (35, 36), length of hospitalization, comorbidities, and mortality.

Drugs used up to the admission and drugs prescribed during hospitalization and discharge were considered separately. BBs were grouped in β1-selective BBs and non-selective BBs, according to β1-receptor selectivity. Indication of use was considered for each molecule. Contraindications to BB use were identified according to the summary of product characteristics (SPCs), for each BB, and patients with contraindicate prescriptions were evaluated. Patients without a BB treatment at admission and discharged with a BB prescription were considered new users; discontinuers and switchers were identified as patients using a BB at admission and discharged without a BB or with a different BB, respectively. Patients using the same BB at admission and discharge were considered continuers. Patients who died during the hospital stay were excluded from the evaluation of drugs prescribed at discharge.

### Statistical Analyses

A descriptive analysis was performed to compare all the characteristics of the study population. All data were reported as absolute and relative frequencies for categorical variables, while medians with interquartile range (Q1–Q3) were calculated for continuous variables. The Barthel and Geriatric Depression Scale was reported as binomial variables. The Kolmogorov–Smirnov test was applied to evaluate the sample distribution. Due to the non-normal distribution of some variables, a non-parametric approach was adopted. The two-tailed Pearson's chi-squared test and the Mann–Whitney *U* test for independent samples were used to compare categorical or continuous variables, respectively. The McNemar test was applied to compare the frequencies of categorical variables between admission and discharge.

To assess the possible influence of all the considered characteristics of the study population on BB prescriptions and β1 selective BB choice, univariate logistic regression models were carried out using patients without BB or with non-selective BB prescriptions as comparators, both in patients affected by HF as a whole and in the subgroup of patients affected by HF and COPD.

All predictors identified in the univariate models were included in a stepwise multivariate logistic regression model (backward elimination procedure, α = 5%). Moreover, all variables that did not result in significant in the univariate analysis, but were considered clinically remarkable after a careful consideration based on current knowledge and clinical expertise, and with a cut-off of alpha error of 0.2 according to the Hosmer–Lemeshow test, were also included (37, 38). Conversely, variables with the same clinically significant and with a plausible collinearity, verified by the Spearman's rank correlation coefficient, were excluded from the multivariate model. Furthermore, in the subgroup of patients affected by HF and COPD, univariate logistic regression models were carried out to evaluate if the BB use influenced the probability of rehospitalization or death within 12 months from the admission.

Odds ratios (ORs) with 95% CIs were calculated for each covariate of interest in the univariate (crude OR) and multivariate (adjusted OR) models. The goodness of fit of the regression models was assessed by the Hosmer–Lemeshow test for adequacy. A *p-*value < 0.05 was considered statistically significant. The statistical analysis was performed using SPSS version 23.0 (IBM Corp., SPSS Statistics, Armonk, NY, USA).

## Results

Of 4,713 patients recorded in the REPOSI register during the years 2010–2016, 942 (20.0%) were affected by HF. Of these, 274 (29.1%) also had COPD and 56 (5.9%) died during hospitalization. Patients with both HF and COPD were mostly men, with greater comorbidity indices (CIRS.S and CIRS.C) and were treated with more drugs, both at admission and at discharge, compared to patients affected by HF alone. In addition, more patients with HF and COPD were concomitantly affected by ischemic heart disease and atherosclerosis or peripheral vascular disease than patients affected by HF alone (Table 1). Heart rate (HR) resulted in <50 bpm in 6 out of 942 patients affected by HF, and exacerbation of COPD was reported as the cause of hospitalization in 25 out of 274 (9.1%) patients affected by HF and COPD.

**Table 1:** Sociodemographic and clinical characteristics of the REPOSI population.

	**HF *N* = 668**	**HF and COPD *N* = 274**	***P*-value**
Gender (M); N (%)	290 (43.4)	172 (62.8)	<0.001
Age, median (Q1–Q3)	82.3 (76.7–86.5)	82.8 (76.7–86.5)	0.830
BMI, median (Q1–Q3)	26.0 (22.9–29.6)	26.4 (23.0–29.8)	0.643
Systolic blood pressure, median (Q1–Q3)	130 (117–140)	130 (115–140)	0.304
Diastolic blood pressure, median (Q1–Q3)	70 (65–80)	70 (70–80)	0.755
Heart rate, median (Q1–Q3)	78 (70–90)	80 (70–90)	0.903
BI >10, N (%)	251 (42.1)	116 (45.0)	0.440
GDS >2, N (%)	115 (20.4)	55 (23.3)	0.352
CIRS_S, median (Q1–Q3)	1.7 (1.5–2.0)	1.8 (1.7–2.1)	<0.001
CIRS_C, median (Q1–Q3)	3.0 (2.0–5.0)	4.0 (3.0–5.0)	<0.001
N° different molecules at admission, median (Q1–Q3)	6 (4–8)	7 (5–9)	<0.001
N° different molecules at discharge, median (Q1–Q3)	7 (5–10)	8 (6–11)	<0.001
Length of hospital stay, median (Q1–Q3)	10 (7–15)	10 (7–14)	0.937
N. of in-hospital mortality	39 (5.8)	17 (6.2)	0.829
Cardiovascular comorbidities			
Hypertension	418 (62.6)	185 (67.5)	0.151
Cardiac dysrhythmias	301 (45.1)	127 (46.4)	0.718
Ischaemic heart disease	187 (28.0)	101 (36.9)	0.007
Cerebrovascular disease	15 (2.2)	8 (2.9)	0.543
Atherosclerosis/Peripheral vascular disease	120 (18.0)	69 (25.2)	0.012

*BMI, body mass index; CIRS_S, Cumulative Illness Rating Scale Severity Index; CIRS_C, Cumulative Illness Rating Scale Comorbidity Index; BI, Barthel Index; GDS, Geriatric Depression Scale*.

Patients affected by HF and treated with at least one BB at admission were 444 (47.1%). Of them, 344 (77.5%) were continuers and 76 (17.1%) discontinued the therapy, while 24 (5.4%) died during hospital stay. At discharge, of 466 patients who were alive and had not previously been treated with BB, 79 (17.0%) new users were identified.

The use of BBs was significantly lower in patients with HF and COPD than in patients affected by HF alone, both at admission and at discharge (admission, 36.9% vs. 51.3%, *p* < 0.001; discharge, 38.1% vs. 51.7%, *p* < 0.001). Furthermore, no significant difference in BB users was observed between admission and discharge in both the HF and COPD affected patients (*p* = 0.864) and in those with HF alone (*p* = 0.999). BBs were used in 4 patients (2 non-selective BBs and 2 β1-selective BBs) admitted because of COPD exacerbation.

The probability of being treated with a BB significantly increased over time in patients affected by HF and ischemic heart disease. Furthermore, the probability of using a BB was significantly lower in patients with HF also affected by COPD (adjusted OR, 95% CI: 0.50, 0.37–0.67; *p* < 0.001) (Table 2). Moreover, in the subgroup of patients with HF and COPD, we observed an increased probability of being treated with a BB if also affected by ischemic heart disease (adjusted OR, 95% CI: 1.90, 1.12–3.23; *p* = 0.017), atrial fibrillation (adjusted OR, 95% CI: 1.96, 1.17–3.28; *p* = 0.011), and chronic kidney disease (CKD) (adjusted OR, 95% CI: 1.68, 1.01–2.80; *p* = 0.047). Conversely, BB use was inversely related to age (adjusted OR, 95% CI: 0.95, 0.92–0.99; *p* = 0.010). Rehospitalizations or deaths within 1 year occurred in 3 and 11 BB users, respectively, while 6 patients were rehospitalized, and 33 died within 1 year among patients not treated with BBs. In detail, the use of BBs reduced the probability of dying within 12 months (OR, 95% CI: 0.38, 0.18–0.78; *p* = 0.009) and the rehospitalizations (OR, 95% CI: 0.15, 0.03–0.91; *p* = 0.040) compared to non-users.

**Table 2:** Factors associated with BB use in patients with HF.

	**Crude OR [CI 95%]**	***P*-value**	**Adjusted OR [CI 95%]**	***P*-value**
Gender (M)	0.82 (0.63–1.06)	0.132	0.92 (0.68–0.24)	0.569
Age	0.98 (0.96–0.99)	0.044	0.98 (0.96–0.99)	0.045
Year of admission			1.08 (1.01–1.15)	0.016
2010	-	-	-	-
2012	1.25 (0.90–1.77)	0.197		
2014	1.67 (1.17–2.37)	0.004		
2016	1.67 (1.15–2.42)	0.007		
BMI	1.00 (0.97–1.02)	0.878		
BI >10	0.90 (0.68–1.18)	0.428		
GDS >2	0.91 (0.65–1.28)	0.592		
CIRS_S	1.08 (0.74–1.58)	0.685		
CIRS_C	1.04 (0.97–1.11)	0.273		
N° different molecules	1.03 (0.99–1.06)	0.058	1.15 (0.98–1.05)	0.357
Length of hospital stay	0.99 (0.99–1.00)	0.208		
Comorbidity				
Hypertension	0.96 (0.73–1.26)	0.770		
Diabetes mellitus	1.13 (0.86–1.48)	0.392		
Atrial fibrillation	1.22 (0.94–1.58)	0.141	1.28 (0.97–1.67)	0.080
Ischaemic heart disease	1.95 (1.46–2.61)	<0.001	2.24 (1.65–3.04)	<0.001
Anemia	1.45 (1.05–1.99)	0.023	1.37 (0.98–1.91)	0.065
CKD	1.19 (0.91–1.56)	0.209		
COPD	0.50 (0.38–0.67)	<0.001	0.50 (0.37–0.67)	<0.001
Neoplasm	0.84 (0.60–1.19)	0.337		
Mood disorders	1.08 (0.76–1.53)	0.683		
Peripheral vascular disease	0.80 (0.57–1.12)	0.186	0.84 (0.59-1.19)	0.321
Gastritis	1.09 (0.75–1.58)	0.667		
Rheumatic disease	1.05 (0.72–1.53)	0.793		
Atherosclerosis	1.04 (0.66–1.62)	0.875		
Cerebrovascular disease	1.25 (0.54–2.92)	0.602		
Benign prostatic hyperplasia	0.69 (0.46–1.03)	0.066	0.67 (0.44–1.02)	0.065

*CI, confidence interval; OR, odds ratio; BMI, body mass index; CIRS_S, cumulative illness rating scale severity index; CIRS_C, cumulative illness rating scale comorbidity index; BI, barthel index; GDS, geriatric depression scale; CKD, chronic kidney disease; COPD, chronic obstructive pulmonary disease*.

The 23.8% and 25.5% of patients with HF and COPD used a non-selective BB at admission and at discharge, respectively (*p* = 0.500). Furthermore, no significant differences were observed in β1-selective BB use between patients with HF and COPD or with HF alone, both at admission (73.2% vs. 76.2%, *p* = 0.538) and at discharge (74.2% vs. 74.5%, *p* = 0.947).

In addition, among patients affected by HF alone, 9 out of 92 users of non-selective BBs at admission (9.8%) switched to β1-selective BBs, while 7 out of 251 users of β1-selective BBs at admission (2.8%) switched to non-selective BBs at discharge. In patients affected by HF and COPD, no patients out of the 24 admitted with non-selective BBs switched to β1-selective BBs, whereas 2 out of 77 users of β1-selective BBs at admission (2.6%) switched to non-selective BBs at discharge.

Among the 79 new BB users at discharge, 61 were affected by HF alone and 18 by HF and COPD. A non-selective BB was chosen as starting treatment in 17 (27.9%) and 5 (27.8%) patients affected by HF alone or HF and COPD, respectively.

The probability of using a β1-selective BB in subjects with HF increased over time and in the presence of hypertension. Conversely, the likelihood of β1-selective BB use was significantly lower in patients with high BI and those with ischemic heart disease. The diagnosis of COPD was not significantly associated with the choice of BB type (adjusted OR, CI 95%: 1.33, 0.76–2.34; *p* = 0.322) (Table 3). Furthermore, no factors significantly influenced the use of β1-selective BB in the subgroup of patients affected by HF and COPD.

**Table 3:** Factors associated with β1-selective BB choice in patients affected by HF and treated with BBs.

	**Crude OR [CI 95%]**	***P*-value**	**Adjusted OR [CI 95%]**	***P*-value**
Gender (M)	0.69 (0.45–1.07)	0.101	0.80 (0.50–1.28)	0.348
Age	1.01 (0.98–1.04)	0.691	0.99 (0.96–1.03)	0.584
Year of admission			1.18 (1.05–1.32)	0.005
2010	–			
2012	1.32 (0.74–2.36)	0.351		
2014	1.11 (0.63–1.96)	0.709		
2016	3.72 (1.78–7.76)	<0.001		
BMI	1.01 (0.97–1.06)	0.588		
BI >10	0.62 (0.40–0.98)	0.041	0.60 (0.38–0.96)	0.033
GDS >2	1.07 (0.59–1.94)	0.824		
CIRS_S	0.79 (0.42–1.48)	0.460		
CIRS_C	0.93 (0.83–1.04)	0.207		
N° different molecules	1.02 (0.95–1.08)	0.610		
Length of hospital stay	1.01 (0.98–1.03)	0.698		
Comorbidity				
Hypertension	1.41 (0.90–2.21)	0.137	1.74 (1.05–2.89)	0.032
Diabetes mellitus	0.75 (0.48–1.17)	0.198	0.81 (0.50–1.32)	0.398
Atrial fibrillation	1.14 (0.73–1.76)	0.568		
Ischemic heart disease	0.56 (0.36–0.87)	0.010	0.51 (0.32–0.81)	0.005
Anemia	1.37 (0.81–2.31)	0.243		
CKD	0.91 (0.58–1.42)	0.671		
COPD	1.02 (0.61–1.71)	0.947	1.33 (0.76–2.34)	0.322
Neoplasm	0.87 (0.47–1.63)	0.669		
Mood disorders	2.02 (1.02–4.01)	0.045	1.47 (0.69–3.15)	0.318
Peripheral vascular disease	0.78 (0.44-1.36)	0.376		
Gastritis	0.89 (0.49–1.61)	0.700		
Rheumatic disease	0.76 (0.42–1.39)	0.371		
Atherosclerosis	0.94 (0.45–1.95)	0.870		
Cerebrovascular disease	1.40 (0.29–6.69)	0.674		
Benign prostatic hyperplasia	0.70 (0.34–1.46)	0.334		

*CI, confidence interval; OR, odds ratio; BMI, body mass index; CIRS.S, Cumulative Illness Rating Scale Severity Index; CIRS.C, Cumulative Illness Rating Scale Comorbidity Index; BI, Barthel index; GDS, Geriatric Depression Scale; CKD, chronic kidney disease; COPD, chronic obstructive pulmonary disease. **HF***.

Patients with HF were treated with 7 different BBs, while patients affected by HF and COPD used 5 different BBs. The most commonly used BB in both groups was bisoprolol at admission and at discharge. Carvedilol was the second most widely used BB in patients with HF and COPD. Furthermore, the use of bisoprolol increased at discharge both in the HF or HF and COPD affected patients (3.10% and 2.81, respectively) as well as the use of carvedilol (2.41% and 1.75%, respectively), while the use of other BBs decreased (Table 4).

**Table 4:** BBs used by patients affected by HF and HF/COPD.

**Molecule**	**Admission *N* = 343**	**Discharge *N* = 325**	**Δ % Discharge-Admission**
Atenolol	25 (7.3%)	18 (5.5%)	−1.75%
Bisoprolol	192 (56.0%)	192 (59.1%)	3.10%
Carvedilol	73 (21.3%)	77 (23.7%)	2.41%
Metoprolol	20 (5.8%)	18 (5.5%)	−0.29%
Nebivolol	14 (4.1%)	13 (4.0%)	−0.08%
Propranolol	8 (2.3%)	3 (0.9%)	−1.41%
Sotalol	11 (3.2%)	4 (1.2%)	−1.98%
**HF and COPD**			
**Molecule**	**Admission** ***N*** **=** **101**	**Discharge** ***N*** **=** **98**	**Δ** **% Discharge-Admission**
Atenolol	7 (6.9%)	5 (5.1%)	−1.83%
Bisoprolol	59 (58.4%)	60 (61.2%)	2.81%
Carvedilol	24 (23.8%)	25 (25.5%)	1.75%
Metoprolol	8 (7.9%)	6 (6.1%)	−1.80%
Nebivolol	3 (3.0%)	2 (2.0%)	−0.93%

Beta-blocker prescriptions were contraindicated in 49 (11.0%) BB users at admission and in 43 (10.9%) BB users at discharge. In particular, 23 (6.7%) and 17 (5.2%) patients with HF alone were treated with a contraindicated BB at admission and at discharge, respectively. In patients concomitantly affected by HF and COPD, contraindicated BB prescriptions resulted in 26 (25.7%) users at admission and 29 (29.6%) at discharge. Carvedilol was the main cause of inappropriate prescriptions in patients with HF and COPD. Moreover, in 11 patients at admission and 13 patients at discharge, the BB was contraindicated because of drug–drug interaction risk.

## Discussion

The use of BBs was evaluated in hospitalized older patients affected by HF and COPD to implement the knowledge about the management of older patients enrolled in the REPOSI database (39, 40). First, HF prevalence was higher than in previous studies (41–43) because of the selection of a different population and diagnostic criteria (44). The prevalence of COPD ranged from 20% to 40% in patients affected by HF. These data were in accordance with our findings, which showed that approximately 30% of patients with HF also had COPD (45–49). Accordingly, the prevalence of HF and COPD increased in men and with age, likely because older patients are generally affected by additional comorbidities (50, 51), compared to patients affected by HF alone (49, 51–54). The high prevalence in men might be due to their increased aptitude to smoke (55); moreover, women have different symptoms and clinical presentation of COPD, and this could lead to an underdiagnosis in women; additionally, the diagnosis of COPD is often mainly difficult in HF with preserved ejection fraction, which most often affects women (56, 57).

Patients with both HF and COPD were treated with multiple drugs both at admission and at discharge than patients affected by HF alone. In fact, the presence of comorbidities could promote a therapeutic approach based on polytherapy, especially in older patients (58, 59); therefore, drug management gets complicated (60, 61).

Despite a clinical and pharmacological rationale suggesting the use of BBs in most of the patients with HF, in this study, fewer than half of the patients were admitted with a BB prescription and, surprisingly, no more BB users were observed after discharge. In fact, <20% of patients started a new BB treatment, while almost 20% of BB users discontinued the therapy after discharge. Moreover, in approximately 10% of BB users, prescriptions were contraindicated because of clinical conditions or drug–drug interactions, and no differences were observed between admission and discharge. Even hospital physicians in internal medicine and geriatrics wards did not properly address the treatment suggested by guidelines.

Although limited to a number of patients, the use of BBs in clinical practice has significantly been associated with a decreased risk of death and rehospitalizations. This confirms once again that beta-blockade is a crucial approach to reduce morbidity and mortality in patients with HF, especially if they are concurrently affected by COPD (6). In fact, all HF guidelines and the Global Initiative for Chronic Obstructive Lung Disease (GOLD) guidelines (23) strongly suggest BB therapy, but β1-selective. In fact, β-receptors blockade decreases the adrenergic hyperactivity occurring in an impaired heart (62) and reduces the risk of arrhythmias and progressive dysfunction of the left ventricle (63). Moreover, BBs improve heart function and, consequently, enhance the pulmonary hemodynamics by relieving symptoms of COPD; long-term use of BBs leads to a reduction in inflammation and pulmonary mucus secretion (64). However, β2-receptors blockade may lead to bronchoconstriction, thus worsening lung function (13); clinical trials highlighted the reduction in forced expiratory volume in the 1st second (FEV1), increased airway hyperresponsiveness, and reduced efficacy of bronchodilator therapy in patients treated with non-cardio-selective BB (17). Otherwise, cardio-selective β1-blockers' use is a safe approach and has a protective effect on all-cause mortality in patients with COPD (65, 66): neither evidence of adverse effects on respiratory function nor influence on efficacy of inhaled β2-agonists on bronchial smooth muscle were observed (67). Nevertheless, the often-unjustified rejection of BB use is a common attitude in clinical practice. The results obtained in this study were in agreement with others that confirmed the underuse of BBs in patients affected by HF and concomitant COPD compared to patients without COPD (10, 53, 68); these data were also relevant at hospital discharge with an increased gap of up to 15% in disfavor of COPD vs. patients with HF alone (69, 70). The limited prescription of BBs in this population is related to the onset of adverse respiratory effects mediated by β2-adrenoceptors blockade in the airways (71, 72).

Once again, the data obtained from the REPOSI register database showed that hospital physicians of internal medicine and geriatrics wards did not modify the treatment with BBs observed at admission; the number of BB users was significantly lower in patients affected by HF and COPD than in patients affected by HF alone; if affected by COPD, the probability of being treated with BBs was about half than that of patients affected by HF alone, and no differences were found at discharge. Moreover, in the subgroup of patients with HF and COPD, the concomitant diagnosis of ischemic heart disease, atrial fibrillation, and CKD was related to a higher use of BBs. A hospital physician prescribed more BBs in patients affected by several conditions for which the use of BBs is approved and is in accordance with guidelines, including ischemic heart disease and atrial fibrillation. On the other hand, the major prescription of BBs in patients affected by CKD is justified by the contraindication of other drugs indicated in HF, such as angiotensin-converting enzyme inhibitors (ACE-i) (73, 74).

Interestingly, the diagnosis of COPD did not influence the choice of BBs in patients with HF, with respect to other data, which suggest a greater use of β1-selective BBs in patients with concomitant COPD than those with HF alone (70). More than 25% of COPD-affected patients treated with a BB were admitted with a non-selective BB prescription, and no differences were observed at discharge. In fact, more contraindicated prescriptions were observed in patients concomitantly affected by HF and COPD than in HF alone, both at admission and at discharge; carvedilol use was the main cause of contraindicate prescriptions in this group of patients. Moreover, about 30% of new BB users were discharged with non-selective BB therapy regardless of COPD.

Current clinical guidelines suggest that metoprolol, bisoprolol, or nebivolol should be preferred in patients with HF and concurrent COPD (23). The most commonly used BB in our study was bisoprolol, followed by carvedilol, with an increase in prescriptions for both at discharge. This finding was different compared to that observed in another observational study, which described a low frequency of bisoprolol prescriptions (68). The use of cardio-selective BBs results in a favorable prognosis and decreased mortality (64, 75). In fact, carvedilol may cause a greater impairment in lung diffusion capacity than cardio-selective BBs due to the alveolar β2 blockade (76). Moreover, the β1-selective bisoprolol showed higher forced expiratory volume in 1 s than carvedilol (77), while carvedilol led to a higher risk of HF hospitalization (78) and, consequently, to COPD exacerbation than cardio-selective BBs (79). Nonetheless, a similar trend in prescriptions of carvedilol was found in several studies that reported that about one-third of patients affected by HF and COPD were treated with carvedilol (80, 81). This could be due to preference for carvedilol in patients affected by HF and milder airflow obstruction (70). However, this choice should be taking into account other comorbidities and co-medications of each patient that physician of internal medicine and geriatric wards had to be considered for the best management of patients affected by HF and concurrent COPD (82).

A discrepancy in prescribing BBs in patients with comorbidities has been observed in our study. The probability of being treated with a BB was significantly higher in patients affected by HF and concurrent ischemic heart disease, but the probability of choosing a β1-selective BB was significantly lower in this group of patients. In other studies, no substantial differences were shown for selective and non-selective BBs in the treatment of subjects with ischemic heart disease (83). Moreover, no clear scientific reasons support this choice; in fact, guidelines suggest the use of a BB without an intrinsic sympathomimetic activity, independently of its selectivity; for this reason, nebivolol should be avoided in patients affected by HF and ischemic heart disease (84, 85). The large number of studies highlighting the efficacy of carvedilol in reducing morbidity and mortality in patients affected by ischemic heart disease, and the poor amount of evidence concerning bisoprolol could have led physicians to choose carvedilol instead of other BBs in this group of patients (86).

In accordance with previous reports (28), hypertension did not increase total BB use in HF-affected patients; however, the probability of choosing a β1-selective BB significantly increased in this group of patients. Physicians preferred β1-selective BBs probably because the β2 adrenal-receptor antagonism reduces antihypertensive effectiveness due to the lack of peripheral vessels vasodilatation and the increased systolic blood pressure variability (87, 88).

In this study, the expected improvement of BB prescriptions after hospitalization was not observed. Largely, the hospital physician in the internal medicine and geriatrics wards takes care of quite complicated patients at the time of admission, especially the older patients affected by different chronic comorbidities, such as cardiovascular diseases (74, 89). In fact, these patients are often treated with several drugs and suffer from a multiorgan damage that could compromise their quality of life. Physicians probably minimize some evaluations by pharmacologists and, therefore, neglect, at least initially, concerns about the best choice of the drugs used during hospitalization. The role of clinical pharmacologist, which is very common in European Hospitals, becomes really important in the multidisciplinary management of patients affected by HF and concomitant COPD, especially for the treatments prescribed at discharge. Several studies have described the possible benefits of the pharmacologist-based intervention in terms of improved quality of life, increased medication appropriateness and adherence, reduction in hospitalization rates, and best management in primary care (90–93). The management of patients with multiple comorbidities and in polytherapy should be implemented not only in the hospital setting but also in the primary care, especially after discharge. Many therapies, including BB use, could be prescribed by general practitioners. Finally, as previously suggested (73, 94), a multidisciplinary approach among hospital physicians, general practitioners, and pharmacologists should be carried out for better drug management and better adherence to guideline recommendations.

The analysis of real-world data in older patients with a high grade of complexity, who are often not included in premarketing studies, is the major strength of this analysis. Moreover, the multicenter design based on the REPOSI register use with a large number of participating centers, which resulted in a wide sample of hospitalized older patients in internal medicine and geriatric wards, supports the novelty of our findings. Current evidence strongly promotes the hypothesis that pharmacological management of patients affected by HF and concomitant COPD should include the identification of other comorbidities and potentially co-medications. Moreover, a personalized care based on a multidisciplinary approach with the close collaboration of hospital physicians and pharmacologists has become really important in order to select the best and the most appropriate therapy. In addition, interventional educational programs may improve physicians' adherence to international guideline recommendations. Furthermore, this study provides an important overview of factors associated with the BB choice and, therefore, the BB use in a large cohort of older adults affected by HF and COPD, hospitalized in internal medicine and geriatric wards, in a long-time study period; moreover, it highlights differences in BB use between admission and discharge, underlining the hospital physicians' therapeutic approach. However, this observational study has some limitations: (i) no specific information on how the diagnosis of COPD was made (GOLD criteria or radiological criteria), and the severity of COPD was not reported in the REPOSI database and we could not analyze how much this affects the prescription, especially primary, of BB or their discontinuation. Similarly, we could not identify patients according to severity and characteristics of HF. (ii) REPOSI concerns only older hospitalized patients; therefore, these findings could not be extended to the general population. Moreover, our findings concern only Italian population, and additional evidences are required to be generalized to other nations.

## Data Availability Statement

The dataset generated for this study will not be made publicly available. Further inquires can be directed to the author SC, salvatore.corrao@unipa.it.

## Ethics Statement

The studies involving human participants were reviewed and approved by Participation was voluntary and all patients provided signed informed consent. REPOSI was approved by the ethics committees of the participating hospitals. The study was conducted according to Good Clinical Practice and the Declaration of Helsinki. The patients/participants provided their written informed consent to participate in this study.

## Author Contributions

VA: conceptualization and writing original draft. FS and SC: writing original draft, writing—review and, editing. MR, MB, and GP: formal analysis. NI, AN, and GN: Validation. CA and GS: investigation. All authors contributed to the article and approved the submitted version.

## Conflict of Interest

The authors declare that the research was conducted in the absence of any commercial or financial relationships that could be construed as a potential conflict of interest.

## Publisher's Note

All claims expressed in this article are solely those of the authors and do not necessarily represent those of their affiliated organizations, or those of the publisher, the editors and the reviewers. Any product that may be evaluated in this article, or claim that may be made by its manufacturer, is not guaranteed or endorsed by the publisher.
